# Dam it's good! DamID profiling of protein‐DNA interactions

**DOI:** 10.1002/wdev.205

**Published:** 2015-09-18

**Authors:** Gabriel N. Aughey, Tony D. Southall

**Affiliations:** ^1^ Department of Life SciencesImperial College London, Sir Ernst Chain BuildingLondonUK

## Abstract

The interaction of proteins with chromatin is fundamental for several essential cellular processes. During the development of an organism, genes must to be tightly regulated both temporally and spatially. This is achieved through the action of chromatin‐binding proteins such as transcription factors, histone modifiers, nucleosome remodelers, and lamins. Furthermore, protein–DNA interactions are important in the adult, where their perturbation can lead to disruption of homeostasis, metabolic dysregulation, and diseases such as cancer. Understanding the nature of these interactions is of paramount importance in almost all areas of molecular biological research. In recent years, DNA adenine methyltransferase identification (DamID) has emerged as one of the most comprehensive and versatile methods available for profiling protein–DNA interactions on a genomic scale. DamID has been used to map a variety of chromatin‐binding proteins in several model organisms and has the potential for continued adaptation and application in the field of genomic biology. *WIREs Dev Biol* 2016, 5:25–37. doi: 10.1002/wdev.205

For further resources related to this article, please visit the WIREs website.

## INTRODUCTION

Regulation of gene expression is primarily coordinated by interactions between proteins and DNA. Understanding the mechanisms by which these proteins mediate transcriptional regulation is fundamental to the study of genetics, cell, and developmental biology. The interactions of myriad DNA‐binding proteins and accessory factors are variable and complex, so the techniques used to interrogate them must be comprehensive and versatile. One such technique is DNA adenine methyltransferase identification (DamID).^1^ In this review, we give an introduction to the theory and application of DamID for detection of chromatin‐protein interactions, contrast with alternative chromatin profiling methodologies, and illustrate the versatility and potential of the technology by highlighting recently published studies in which DamID has been used to provide insights into diverse biological systems. We also discuss recent technical developments in the implementation of DamID and speculate on novel uses for which DamID may be adapted.

## DamID: THEORY, IMPLEMENTATION, AND TECHNICAL CONSIDERATIONS

**Figure 1:**
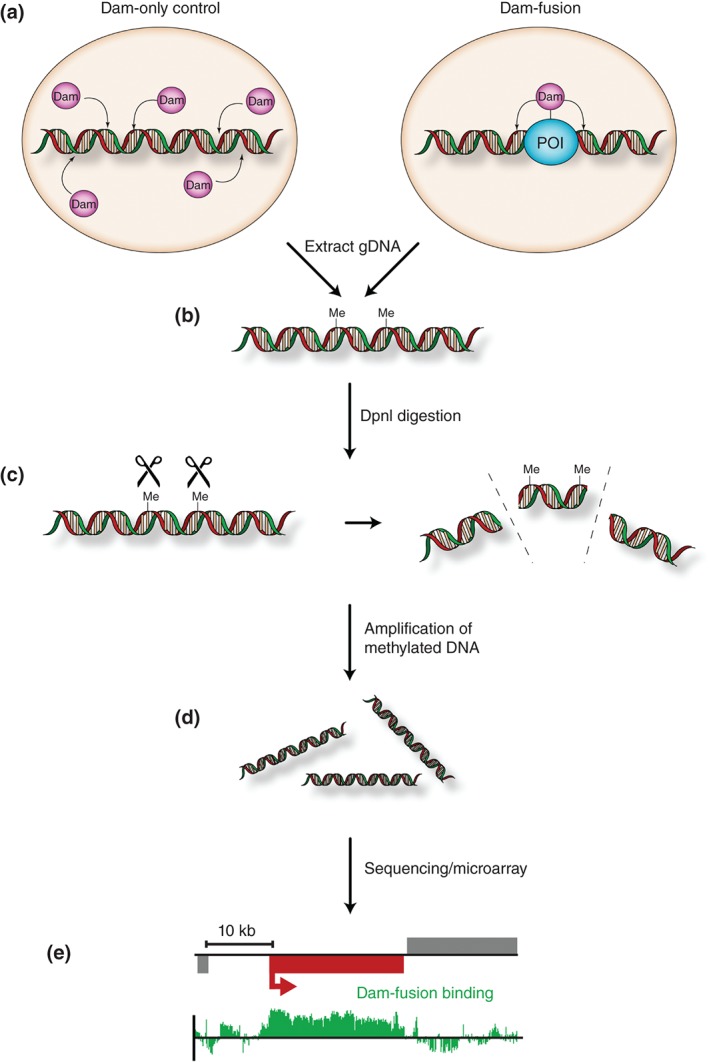
Schematic illustrating DNA adenine methyltransferase identification (DamID) experimental pipeline. (a) Dam only or Dam fused to a protein of interest (POI) (blue) is expressed in a suitable cell type or transgenic organism. (b) Genomic DNA is extracted. DNA obtained includes N6‐adenine methylation sites (Me) catalyzed by Dam. (c) Genomic DNA is digested by the methylation sensitive restriction enzyme, DpnI. (d) Digested fragments are amplified by polymerase chain reaction (PCR). (e) Representative output indicating chromatin binding of a protein of interest at an individual locus. Vertical bars indicate the log_2_ ratio of Dam‐fusion/Dam only.

Methylation is one of the most common covalent modifications of DNA in almost all organisms.^2^ However, differences exist between the methylation of DNA in prokaryotes and eukaryotes, which are exploited in DamID. Methylation of adenine is widespread across many bacterial phyla, but is thought to be largely absent in eukaryotic cells, (although low levels of adenine methylation have recently been reported in *Drosophila*, *Caenorhabditis elegans*, and *Chlamydomonas* 3, 4, 5). The *Escherichia coli* DNA adenine methyltransferase catalyzes the addition of a methyl group to the N6 position of adenine in the sequence, GATC.^6^ DamID relies on expression of *E. coli* Dam as a fusion protein with a chromatin interacting protein of interest (e.g., a transcription factor). Dam is recruited to specific loci by virtue of being tethered to a protein with an affinity for a particular sequence or chromatin environment, and is able to deposit methyl groups on nearby GATC sequences. After extracting methylated DNA from transgenic cells, the methylation sensitive restriction endonuclease, DpnI, can be used to fragment the genomic DNA. Methylated sequences may then be amplified by PCR and detected by various sequencing or array‐based methods to produce a map (chromatin profile) of loci at which the protein of interest has been in close proximity to, during the period in which the Dam‐fusion was expressed (Figure 1).

An important consideration when designing a DamID experiment is choosing the promoter used to express the Dam‐fusion protein. When expressed at high concentrations, methylation levels may be saturating, thus making it difficult to determine *bona fide* binding sites. Furthermore, Dam expression may be toxic to the cell, resulting in experimental artifacts.^1^, ^7^ To increase the signal to background ratio, Dam is typically expressed at very low concentrations. Suitable levels of Dam expression have been achieved by taking advantage of minimal ‘leaky’ expression from inducible promoters. For example, the heat shock inducible *hsp70* promoter in *Drosophila* is often used without heat shocking the experimental flies or cells, resulting in almost undetectable levels of Dam, which nevertheless yield reproducible methylation profiles.^1^ Similarly, leaky expression from non‐induced inducible ecdysone promoters has been used for DamID in mammalian cells.^8^

When expressing a Dam‐fusion protein, a high level of background methylation is typically observed due to the association of Dam with non‐native target loci. To accurately distinguish binding events from this background signal, it is important to compare the methylation profile obtained with an appropriate control. This is usually achieved by expression of Dam alone (i.e., not fused to a DNA‐binding protein). The methylation at a particular loci is often expressed as the ratio of methylation for Dam‐fusion: Dam, thereby, normalizing the background methylation.

Since its inception by Bas Van Steensel and Steven Henikoff in 2000, DamID has been used to profile chromatin interactions in multiple organisms. The technique was first demonstrated in *Drosophila*, which remains the model in which DamID has been most extensively applied.^1^, ^9^ However, DamID has also been widely used to investigate chromatin biology in mammalian cells,^8^ plants,^10^, ^11^, ^12^ yeast,^13^, ^14^, ^15^ and *C. elegans*.^16^, ^17^, ^18^ In theory, DamID can be adapted for any organism in which it is possible to produce transgenic cell lines or animals. Detailed protocols for DamID in various cell types have been published.^8^, ^11^, ^19^

## DamID VERSUS ChIP

When preparing to interrogate chromatin associations of a protein of interest, there are several experimental approaches which can be considered, each with relative advantages and disadvantages (Table 1).^20^ For assessing chromatin binding on a global scale, the leading alternative to DamID, is chromatin immunoprecipitation (ChIP).^21^ The ChIP technique relies on chemically crosslinking DNA and proteins, followed by shearing of DNA into fragments, typically by sonication. An antibody targeting the protein of interest is then used to extract protein–DNA complexes by immunoprecipitation, and the DNA sequences determined by hybridization (ChIP‐chip) or sequencing (ChIP‐seq) methods.^22^ ChIP and DamID may both be used to generate genomic DNA‐binding profiles; however, each technique may be more suitable than the other for a particular application. Methylation by Dam has been shown to extend from any given target locus to a distance of up to 5 kb,^1^, ^9^ therefore, the resolution of DamID data can be lower than that of ChIP. However, several studies have compared the results of DamID to ChIP for the same protein and have demonstrated highly similar results from either technique.^7^, ^23^, ^24^, ^25^, ^26^ Therefore, DamID may be applicable in many instances where ChIP would normally be used unless very high resolution is required. Improvements to the ChIP technique (Chip‐exo) have recently been developed, which allow binding data at single‐nucleotide resolution to be obtained.^27^, ^28^

**Table 1:** Comparison of the Relative Advantages and Disadvantages of DNA adenine methyltransferase identification (DamID) Compared with chromatin immunoprecipitation (ChIP)

	ChIP	DamID
Specific reagents required	Antibody with good specificity and high affinity.	Transgenic cells expressing Dam‐fusion protein of interest.
Resolution	High resolution.	Methylation depends on the distribution of GATC in the genome. Resolution still comparable to ChIP.
Applicable organism	Any organism for which high‐affinity antibody can be obtained.	Any genetically tractable animal or cell type.
Detection of post‐translational modifications	Possible with appropriate antibody.	Not possible.
Tissue‐specific profiling	Requires physical separation of cells or nuclei.	Dam‐fusions can be expressed in a tissue‐specific manner.
Detection of long range or transient interactions	Not possible due to specific binding required.	Methylation of nearby or transiently Dam‐associated sequences is possible.
Requires ‘fixing’ of samples	*In vitro* technique. Requires formaldehyde crosslinking of samples.	Methylation occurs *in vivo*. DNA can be extracted from unfixed or even live cells.
Temporal resolution	Limited only by time taken for fixing (minutes).	Dam must be expressed for several hours.
Isoform specificity	ChIP antibodies may bind to multiple isoforms of the same protein.	A specific sequence must be expressed; therefore, binding of only one isoform is assayed.
Proteins expressed at low levels	May be difficult to purify low expressed proteins with ChIP antibody.	Dam concentration is independent of endogenous protein levels. (Dam‐fusions have to be expressed at very low levels).

One of the main drawbacks of ChIP, in comparison to DamID, is that it requires a highly specific antibody that binds with high affinity to the protein of interest.^29^ Such antibodies can be difficult or expensive to produce. Furthermore, the binding characteristics of each antibody will be unique, meaning that each ChIP experiment must be optimized to ensure appropriate binding and precipitation of DNA sequences is achieved. In contrast to ChIP, no specific reagents are required for a DamID experiment, although transgenic cells or organisms expressing the Dam‐fusion protein of interest must be obtained. This means that following validation of the successful construction of a Dam‐fusion expression construct, the experimental procedures will be identical for each protein assayed. Recent studies have taken advantage of this fact, enabling increased experimental throughput so that multiple interaction profiles may be generated.^30^ Owing to the necessity of creating transgenic cells for DamID, the technique is not applicable for studies in the small minority of model organisms in which transgenesis is not yet possible.

DamID and ChIP contrast in their relative suitability depending on the temporal expression or abundance of the protein of interest. If detecting interactions between DNA and a protein thought to be present at very low abundance, it may be difficult to obtain sufficient binding and purification even with a verified ChIP‐grade antibody. As Dam‐fusion proteins are required to be expressed at extremely low concentrations anyway, low endogenous protein abundance is not an impediment to DamID. For highly expressed proteins, either technique may be appropriate. In contrast, if the experiment requires profiling for a very short‐time interval, ChIP may be a more suitable choice, as Dam‐fusions are typically expressed for a minimum of several hours to achieve robust methylation. These differences in temporal suitability emphasize a fundamental difference between the two protocols, namely that ChIP measures where a protein is bound at any one instance, whereas DamID gives an indication of where a protein has previously been in proximity to the DNA sequence, even if only transiently.

It is possible to use both ChIP and DamID to generate chromatin interaction profiles for individual cell or tissue types of a whole organism. However, for ChIP, this requires separation of the cells or nuclei of interest from the starting material, often using flow cytometry or dissection, both of which are time‐consuming processes. In contrast to this, genetic techniques can be exploited to give Dam methylation only in a tissue of interest, negating the need for cell separation (see ‘Cell type‐specific profiling with DamID’ subheading).^7^

Interpretation of data obtained from any genome‐scale experiment usually requires careful and considered implementation of bioinformatic techniques, specifically tailored to the experiment in question. DamID and ChIP are no exception to this. As ChIP is a longer established technique, the computational tools used to analyse ChIP data are subsequently better developed than for DamID. Furthermore, the majority of studies reporting DamID data have used microarray‐based approaches, while DamID‐seq has only been more widely adopted recently. However, computational pipelines have recently been published for the analysis of DamID‐seq data.^31^, ^32^ Both DamID and ChIP are powerful techniques for determining protein interactions with chromatin. While similar data may be obtained with both methods, the user should be careful to bear in mind the strengths and limitations of each approach. Owing to the differences in the experimental methodologies, DamID and ChIP may be subject to specific artifacts. Therefore, use of DamID and ChIP as complementary approaches can yield higher confidence results than either approach alone. Several studies have used ChIP and DamID as orthogonal approaches to validate *bona fide* chromatin interactions.^24^, ^25^, ^26^

## USING DamID TO UNDERSTAND TRANSCRIPTIONAL REGULATION

**Figure 2:**
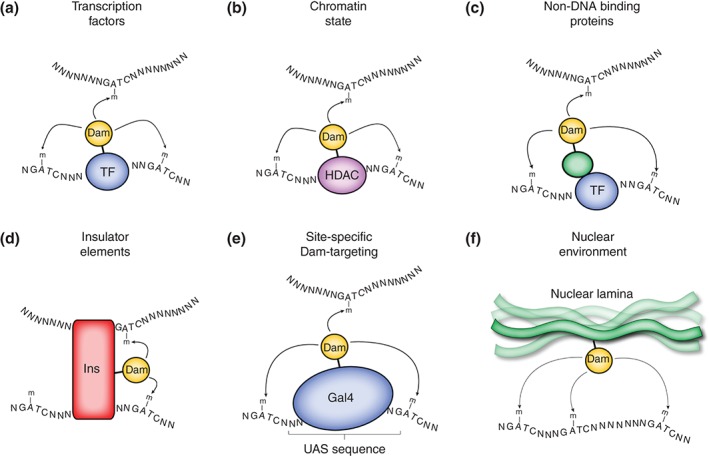
DNA adenine methyltransferase identification (DamID) applications. (a) Dam fused to a transcription factor (TF) with a known binding site can be used to methylate proximal DNA sequences in both *cis* and *trans*. (b) Dam fused to a histone modifier (e.g., a histone deacetylase—HDAC) or chromatin remodeler can be used to give an indication of chromatin state. (c) Dam fused to proteins which do not directly interact with DNA can methylate proximal loci. (d) Dam fused to an insulator protein will methylate all proximal and interacting sequences. (e) Targeting of Dam in a locus‐specific manner using sequence‐specific gene targeting tools, e.g., Gal4/upstream activation sequence (UAS) can be used to detect methylation in *trans*. (f) Dam‐fusions with proteins that make up the nuclear environment (e.g., lamins), can be used to determine interactions with the nuclear lamina or other nuclear compartments.

Of all the protein–DNA interactions that occur, the interaction between transcription factors and promoter or enhancer regions of genes is among the most studied, due to the fundamental role of these proteins in transcriptional regulation. DamID has been extensively utilized to uncover the mechanisms of transcriptional regulation by transcription factor binding in multiple organisms and cell types. DamID has been used to produce chromatin‐binding profiles for a diverse range of transcription factors with various physiological roles (Figures 2a).^33^, ^34^, ^35^, ^36^, ^37^ Owing to the relative simplicity in performing a DamID experiment, chromatin profiles for multiple transcription factors have often been reported, allowing for the generation of high‐resolution transcriptional networks.^24^, ^38^, ^39^ As well as detecting individual novel target loci for these transcription factors, this approach allows for some insight into synergistic or redundant transcription factor binding, and *de novo* identification of important genes in various biological processes. Orian et al., have used this approach to generate chromatin profiles for the Myc/Mnt/Mad network of transcription factors, showing that these factors interact with around 15% of all coding regions.^39^ Furthermore, comparison of binding profiles indicated that Max levels influence the binding of Myc but not Mnt. A similar approach has been taken to investigate chromatin binding of several key transcription factors involved in neural development.^38^ In this study, integration of multiple chromatin profiles into a transcriptional network was used to identify binding of multiple transcription factors at individual loci, to predict important genes involved in neural development. The binding of unrelated transcription factors has also been compared using DamID to gain an understanding of the targeting mechanisms of transcription factors to DNA. In one study, seven transcription factors with diverse physiological roles were shown to frequently co‐localize to the same loci in the *Drosophila* genome, demonstrating the presence of transcriptional ‘hotspots’ at which many proteins may be recruited independently of their DNA‐binding specificity.^24^ The question of transcription factor binding conservation has also been addressed using DamID. Carl and Russell profiled the binding of the transcription factors Dichaete and SoxNeuro in four different *Drosophila* species.^40^ They found that the regulatory networks driven by Dichaete and SoxNeuro are in general conserved across these species; however, they also found that binding site turnover is widespread and linked to phylogenetic distance.

Despite intense study, the manner in which DNA‐binding proteins, such as transcription factors, interact with DNA is little understood in many cases. The fact that all the components needed for a DamID experiment are encoded within the experimental organism allows for the effects of precise manipulations of either the DNA‐binding protein, or target sequences of interest, to be monitored. For example, a Dam‐fusion protein may be expressed in an organism in which the consensus site at a particular locus has been modified, in order to determine the effect of specific mutations on transcription factor binding. Alternatively, mutations can be made in the sequence for the DNA‐binding protein itself to determine which specific residues or domains are required for interactions with genomic loci or consensus motifs. The feasibility of this approach has been demonstrated by Vogel et al., who monitored the effect of genome‐wide chromatin binding when specific point mutations were introduced to CBX1, a human orthologue of heterochromatin protein 1 (HP1).^41^ Similarly, mapping of Bicoid (Bcd)‐targeted sites in the *Drosophila* genome, using a truncated form of the protein lacking a functional DNA‐binding domain, has been shown to have no effect on its targeting to genomic hotspots, indicating that recruitment of Bcd by external factors is sufficient for the interaction of Bcd with some loci.^24^

## USING DamID TO INVESTIGATE CHROMATIN STATES

Chromatin can exist in a variety of configurations within the nucleus, defined by histone occupation or post‐translational modifications, which are thought to closely correlate with the regulation of gene expression. Traditionally, these regions of chromatin have been broadly categorized into euchromatin and heterochromatin (transcriptionally active and repressive, respectively).^42^, ^43^ Chromatin profiling techniques such as DamID have helped to reveal how these regions are defined and have contributed substantially to our understanding of their biological function.

DamID with proteins known to be involved in chromatin structure has been employed extensively to understand how chromatin regions correlate with their underlying DNA sequences (Figures 2). One such experiment investigated the distribution of H1 ‘linker’ histones throughout the genome. Human somatic cells have five reported histone H1 variants; however, the reason for this heterogeneity is not well understood. By profiling these H1 subtypes with DamID, it was shown that enrichments of individual H1 variants were correlated with genomic features such as CpG islands or active/repressive domains.^44^ HP1 is one of the most well‐characterized protein constituents of heterochromatin. DamID profiles of genome‐wide HP1 binding have been generated in *Drosophila* and *Arabidopsis* to help elucidate the mechanisms of heterochromatin formation.^10^, ^45^ HP1 was shown to associate preferentially with genes flanked by repeat regions in *Drosophila*. Interestingly, this study revealed that HP1 is significantly associated with X chromosomes in males only. In *Drosophila*, dosage compensation upregulates transcription from the male chromosome, therefore, these data indicated a previously unknown role for HP1 in the activation of gene expression. The association of HP1 with transcriptionally active genomic loci has subsequently been more extensively characterized using DamID.^46^ DamID profiling of HP1 has also been used to study the mechanisms of heterochromatin spreading in position‐effect variegation.^47^

DamID can also be used to profile chromatin binding of proteins known to induce chromatin modifications such as acetylation or methylation of histones. Such an approach can give an indication of genomic regions that have undergone chromatin modifications, (although this may not be representative of the chromatin state at the time of DNA extraction). DamID has been used to generate binding profiles for a histone deacetylase (HDAC4) in rat cardiomyocytes.^48^ Comparison of these data with expression profiles for genes in the same cell type as well as ChIP was used to validate HDAC occupancy at these loci. Similarly, MBD3, a component of the nucleosome remodeling and histone deacetylase complex has had its chromatin‐binding profile determined by DamID in human cancer cell lines, thereby, indicating regions in which nucleosome remodeling activity may be active.^26^ Polycomb group proteins are a well‐known group of proteins known to facilitate chromatin remodeling that are important for the repression of transcription at many genes.^49^ DamID profiling of the polycomb repressive complexes PRC1 and PRC2 in *Drosophila* identified extensive regions of polycomb binding and implicated polycomb mediated repression in several previously unidentified biological processes.^23^

In a recent study, DamID was used to profile binding for over 50 different proteins known to be associated with chromatin in *Drosophila* cultured cells.^30^ From these data, it could be seen that chromatin can be broadly categorized into five principle types based on association with different groups of proteins. Furthermore, this approach was then extended to identify previously unknown constituents of chromatin through screening more than one hundred candidate genes for reproducible methylation profiles. Using this approach, 42 novel chromatin components were identified, thereby, considerably expanding the known complement of chromatin proteins.^50^

Although DamID is predominantly used to target methylation to specific genomic loci, the relative promiscuity of Dam has also been exploited to characterize chromatin states more broadly. Sha et al. have used Dam expression in the absence of a fusion protein to probe accessible regions of the *C. elegans* genome.^51^ This study demonstrated that untethered Dam could reliably be used to highlight regions of chromatin with high nucleosome coverage as indicated by the lack of methylation, presumably due to inaccessibility to Dam.

## USING DamID TO UNDERSTAND NUCLEAR ARCHITECTURE AND CHROMATIN DYNAMICS

The spatial organization of chromatin within the nucleus is highly regulated in order to achieve efficient packaging of DNA and to mediate transcriptional regulation. DamID has proved to be an invaluable resource in understanding how chromatin is arranged with respect to itself and the nuclear environment. Dam‐fusion proteins have been shown to have the ability to methylate GATC sequences up to 5 kb away from a Gal4‐targeted locus.^1^ The ability of Dam to methylate DNA across relatively large distances has been exploited by several recent studies to understand the organization of chromatin within the nucleus.^52^ It has been shown that the long‐range methylation of sequences on a strand to which Dam is targeted (*cis*‐methylation) is also seen on distinct DNA strands at sequences in close proximity to a Dam‐targeted locus (trans‐methylation).^52^, ^53^ Consequently, DamID has emerged as a versatile alternative technique to chromatin conformation experiments such as 3C to detect looping of chromatin and local DNA interactions (Figures 2).

In order to examine the nature of such chromatin looping, it may be necessary to target Dam to a specific locus. This can be achieved by a number of different strategies. First, Dam‐fusions can be created for proteins known to bind at a specific locus. Although this approach may appear straightforward, it is unlikely that any given transcription factor, insulator, or other chromatin‐binding protein will bind to a single locus. Therefore, this strategy may be applicable if *trans*‐interacting loci are already known, but not for the identification of novel interacting loci. Alternatively, Dam may be tethered to a specific locus by the manipulation of the sequence of interest to include a binding site for an exogenous DNA‐binding protein. This method has the advantage of being able to target one distinct locus, as well as providing the option of a control in which no exogenous sequence has been inserted for the Dam‐fusion to bind.

In *Drosophila* this has been demonstrated using the well‐characterized yeast transcription factor, GAL4. In this case, a GAL4 upstream activation sequence (UAS) was inserted into the genome adjacent to the site of interest to which the Dam‐GAL4 fusion protein could bind (Figures 2e). This technique has been used to identify regulatory interactions in the *Drosophila* bithorax complex. Dam‐GAL4 was targeted to the *Fab‐7* region and resulted in strong methylation at the *Abd‐bm* locus, situated around 35 kb downstream, thereby, demonstrating that the chromatin looping occurs in this region to regulate gene expression.^53^ *Drosophila* lines containing transposon insertions that include UAS at multiple loci (EP elements) are readily available,^54^ therefore, it is feasible that this approach could be scaled up to produce genome‐scale, high‐resolution maps of chromosome looping. The TetR system has been used for similar effect in *Saccharomyces cerevisiae*. Fusion of Dam to the DNA‐binding domain of TetR was used to target Dam to Tet operators inserted at the *HML* locus. In this study, high levels of methylation were detected in *trans* at telomeric sequences, including those of different chromosomes, indicating a spatial relationship between the *HML* locus and telomeres within yeast nuclei.^15^

Recent advances in genome‐targeting technologies may provide an even more efficient way to target Dam to specific loci. Recombinant proteins with affinity for specific loci, such as TALEs (transcription activator‐like effectors) may now be relatively easily produced.^55^ It is conceivable that Dam fused to a TALE would result in targeted methylation similar to that described for Gal4‐Dam, but with the advantage of not having to manipulate the underlying DNA sequence. In such a system in which the endogenous locus is intact would reduce the risk of experimental artifacts, and allow for more subtle manipulations of the region to assess the effect on chromatin looping or enhancer activity.

DamID has also been used to understand the interactions between chromatin and its surrounding environment. By fusing Dam with components of the nuclear lamina, it has been possible to determine the sequences that are located at the nuclear periphery or interact with the nuclear envelope (Figures 2f).^56^ This approach has led to the identification of lamina‐associated domains (LADs) that are large, well‐defined domains, typically containing genes in which transcription is repressed, in both human and *Drosophila* cells.^57^ Similarly, DamID has been used to identify regions of chromatin in close proximity to nuclear pore complexes (NPCs) by expression of nucleoporin‐Dam‐fusions.^58^ NPCs were found to associate predominantly with transcriptionally active genes and were subsequently shown to regulate transcription of those genes.

Chromatin is known to be dynamic within the nucleus, with genomic regions undergoing dramatic spatial rearrangements concomitant with cell differentiation or transcriptional state.^59^ These dynamic changes in chromatin architecture are known to be crucial for regulating gene expression. The DamID technique has been adapted to enable the imaging of chromatin dynamics *in vivo*.^60^ This has been achieved by the expression of a truncated DpnI restriction endonuclease (with no catalytic activity) fused to enhanced green fluorescent protein (eGFP). The eGFP‐DpnI truncation recognizes and stably binds to methylated GATC sequences, which can then be visualized *in vivo*. This technique has been used to track the movements of LADs through mitosis in a single cell in real time, providing valuable insights into chromatin dynamics throughout the cell cycle.

## CELL‐TYPE‐SPECIFIC PROFILING WITH DamID

**Figure 3:**
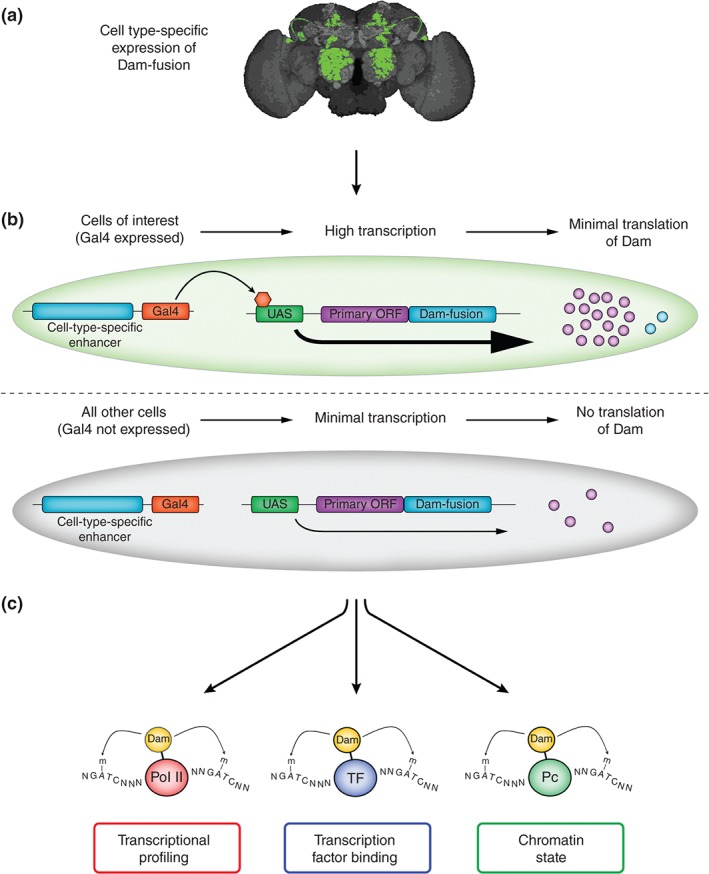
Schematic illustrating cell type‐specific chromatin profiling with targeted DamID (TaDa). (a) Dam can be expressed in individual cell populations e.g., individual neuronal populations (green) within the CNS (gray). (b) In cells in which Gal4 is expressed (green), the bicistronic transcript is expressed at high levels, leading to low‐level translation of the secondary open reading frame (ORF) containing the Dam‐fusion. Where Gal4 is not expressed (grey), there is minimal expression of the bicistronic transcript. (c) Multiple uses for Tada depending on Dam‐fusion protein. RNA polymerase II (PolII)—Dam‐fusions can be used to generate a transcriptional profile for an individual population of cells. Transcription factor (TF)—Dam‐fusions may highlight differences in TF binding between cell types. Chromatin proteins (e.g., Polycomb—Pc), can be used to highlight differences in chromatin state between cell types.

Multicellular organisms are typically comprised of a very diverse set of cell types. In order to properly understand development and cellular function in the adult organism, the gene regulatory mechanisms need to be interrogated at a cell type‐specific level. Until recently this was not possible with DamID, due to the toxicity of ectopic Dam expression in most cell types. Previous DamID experiments have typically been conducted in whole organisms using a low level‐expressing basal heat shock promoter. However, recent studies in *Drosophila* have demonstrated that the low‐level induction of Dam in a tissue specific and temporally controlled manner can be achieved to produce DNA‐binding profiles with spatial and temporal resolution without the need for cell purification (Figures 3).^7^

In order to drive the cell‐specific expression of Dam at sufficiently low levels (to prevent methylation‐induced toxicity or saturating methylation levels), an expression system was developed in which Dam‐fusion proteins are encoded as a secondary open reading frame (ORF) in a bicistronic expression construct downstream of a UAS enhancer. This system takes advantage of the phenomenon of ribosome reinitiation to express the product of the secondary ORF at very low levels.^61^ In the absence of transcriptional activation, basal transcription results in low levels of translation from the upstream ORF, with negligible translation of the Dam‐fusion from the secondary ORF. When combined with a GAL4 driver line, translation levels are drastically increased from the primary ORF, whereas translation of the Dam‐fusion occurs at low levels, enough to result in tissue‐specific methylation of target sequences, without causing toxicity. This method has been named Targeted DamID (TaDa).^7^ Although TaDa has not yet been demonstrated in species other than *Drosophila*, the principles are likely to apply to other model systems.

The ability to easily perform DamID experiments in a cell type‐specific manner has drastically increased the scope of experiments possible to help understand chromatin biology in multicellular species. One particularly useful application of TaDa that has been demonstrated is its ability to profile the transcriptional state of specific cells. For this purpose, Dam was fused to the core subunit of RNA polymerase II (Pol II) and expressed in two separate populations of *Drosophila* neural stem cells in order to assay the genome‐wide occupancy of Pol II on chromatin.^7^ Significant differences in methylation at individual loci was observed in these cell types, which were subsequently validated by orthogonal methods, indicating that TaDa is a powerful method for assessing gene expression in a cell type of interest, as an alternative to methods that require cell isolation before RNA‐seq.

Aside from transcriptional profiling, TaDa also has the potential to be employed for a range of novel applications. The binding of transcription factors to DNA is likely to be highly context specific, therefore, data produced from DamID experiments conducted in whole organisms may mask variation in transcription factor binding in individual cell or tissue types. TaDa may be employed to assess the extent to which there exists diversity of binding for ubiquitously expressed transcription factors across cell types. It is likely that the wealth of enhancer trap Gal4 lines available in *Drosophila* will allow for extensive characterization of tissue‐specific transcription factor occupancy.

## CONCLUSION

Advances in chromatin profiling technologies have greatly improved our understanding of many fundamental biological processes including transcriptional regulation, DNA replication, and cell division. The use of DamID for profiling the interaction of proteins with chromatin has proved to be a valuable resource in the molecular biologist's toolkit, either as a complementary technique to existing technologies or as a powerful discovery methodology in its own right. The examples highlighted here illustrate just some of the applications for which DamID may be utilized, and it is likely that novel uses will emerge as the technology matures.

Despite the many advantages of DamID, the technology is not without shortcomings which may have prevented DamID from becoming as widely used as its alternatives. For example, the lack of resolution in comparison to ChIP may put off some researchers. However, the simplicity, versatility, and potential for increased throughput may be sufficient to convert even the most ardent ChIP enthusiast. The fact that DamID requires handling of only DNA and not relatively unstable or difficult to handle substances such as RNA or protein, will also be appealing to researchers with limited proteomics experience. Furthermore, the ever decreasing cost of sequencing makes DamID accessible to more and more researchers. It is likely that the next few years will yield developments in the methodology and application of DamID, some of which will come from unexpected directions. However, several improvements to the method are desirable and are therefore likely to emerge due to necessity. First, it is probable that DamID will be adapted for use in even more diverse model systems, especially considering recent advances in genome editing technologies such as CRISPR/Cas9. Currently, DamID has only been demonstrated in mammalian cell lines; however, the development of DamID in mice, especially in a cell type‐specific manner, is an attractive prospect for understanding mammalian chromatin biology. TaDa also has the potential to be applied to organoids derived from human stem cells, which could be a powerful tool for investigating development and regulation of gene expression in human tissues. Second, improvements to the resolution of DamID will be sought after by some researchers, especially those who wish to explore the precise nature of interaction of DNA‐binding proteins with their underlying DNA sequences. It is conceivable that modifications of the Dam enzyme itself could result in more well‐defined methylation profiles. Some progress has been made in this area with the development of DamIP, a technique in which expression of a tethered Dam methylase is combined with immunoprecipitation using an antibody targeting N6 methylated adenine.^62^, ^63^, ^64^ Crucially, this technique is used in combination with a mutant form of Dam (DamK9A) in which both specificity and activity of the enzyme are increased,^65^, ^66^ resulting in methylation at a greater number of proximal loci. Lastly, more highly sensitive detection methods would be useful for researchers studying chromatin dynamics at single‐cell resolution. This may be achieved through a combination of improvements to the DamID technology itself and advances in next generation sequencing.

The ‘proximity labeling’ approach at the heart of DamID has already inspired the development of similar methods in other areas of cell biology such as BioID. In BioID, a protein of interest is tagged with a promiscuous biotin ligase (analogous to Dam in DamID), which is able to biotinylate nearby proteins, which can then be detected using proteomic methods.^67^ It is possible that the proximity labeling approach will be adapted in the future to profile other biological molecules of interest. With an ever increasing number of researchers asking more hypothesis driven questions about how genes are expressed and regulated in tissues and specific cell types, the elegance and utility of DamID, and its derivative TaDa, will undoubtedly become a popular tool for developmental biologists, physiologists, and stem cell biologists alike.
